# Activation of Toll-like receptors by *Burkholderia pseudomallei*

**DOI:** 10.1186/1471-2172-9-46

**Published:** 2008-08-08

**Authors:** T Eoin West, Robert K Ernst, Malinka J Jansson-Hutson, Shawn J Skerrett

**Affiliations:** 1 Department of Medicine, University of Washington, Seattle, Washington, USA

## Abstract

**Background:**

Melioidosis, a lethal tropical infection that is endemic in southeast Asia and northern Australia, is caused by the saprophytic Gram-negative bacterium *Burkholderia pseudomallei*. Overall mortality approaches 40% yet little is known about mechanisms of host defense. Toll-like receptors (TLRs) are host transmembrane receptors that recognize conserved pathogen molecular patterns and induce an inflammatory response. The lipopolysaccharide (LPS) of Gram-negative bacteria is a potent inducer of the host innate immune system. TLR4, in association with MD-2, is the archetype receptor for LPS although *B. pseudomallei *LPS has been previously identified as a TLR2 agonist. We examined TLR signaling induced by *B. pseudomallei*, *B. pseudomallei *LPS, and *B. pseudomallei *lipid A using gain-of-function transfection assays of NF-κB activation and studies of TLR-deficient macrophages.

**Results:**

In HEK293 cells transfected with murine or human TLRs, CD14, and MD-2, heat-killed *B. pseudomallei *activated TLR2 (in combination with TLR1 or TLR6) and TLR4. *B. pseudomallei *LPS and lipid A activated TLR4 and this TLR4-mediated signaling required MD-2. In TLR2^-/- ^macrophages, stimulation with heat-killed *B. pseudomallei *augmented TNF-α and MIP-2 production whereas in TLR4^-/- ^cells, TNF-α, MIP-2, and IL-10 production was reduced. Cytokine production by macrophages stimulated with *B. pseudomallei *LPS or lipid A was entirely dependent on TLR4 but was increased in the absence of TLR2. TLR adaptor molecule MyD88 strongly regulated TNF-α production in response to heat-killed *B. pseudomallei*.

**Conclusion:**

*B. pseudomallei *activates TLR2 and TLR4. In the presence of MD-2, *B. pseudomallei *LPS and lipid A are TLR4 ligands. Although the macrophage cytokine response to *B. pseudomallei *LPS or lipid A is completely dependent on TLR4, in TLR2^-/- ^macrophages stimulated with *B. pseudomallei, B. pseudomallei *LPS or lipid A, cytokine production is augmented. Other MyD88-dependent signaling pathways may also be important in the host response to *B. pseudomallei *infection. These findings provide new insights into critical mechanisms of host defense in melioidosis.

## Background

Melioidosis is an endemic and poorly understood infectious disease in much of the tropical world; it is particularly prevalent in east Asia and northern Australia. The disease accounts for 20% of community-acquired sepsis in parts of northeast Thailand. Despite antibiotic treatment, mortality rates approach 40% [1]. The causative organism, *Burkholderia pseudomallei *(*Bp*), is a Gram-negative environmental saprophyte. Aerosol or transcutaneous infection results in an extensive range of disease – from chronic, relapsing illness with abscess formation to fulminant pneumonia and septicemia [2]. The lung is the most commonly affected organ. Concern about the use of *Bp *as a bioweapon has led to its classification as a CDC Category B pathogen. While there are several known predisposing factors to clinical infection, such as diabetes, renal or liver disease, alcoholism, or immunosuppression [3], relatively little is known about mechanisms of host susceptibility.

Innate immune signaling mechanisms comprise the front line of host defense against infection. Toll-like receptors (TLRs) are transmembrane receptors in the IL-1 receptor superfamily that are activated by conserved pathogen-associated molecular patterns and result in nuclear transcription factor (NF)-κB translocation and induction of a pro-inflammatory response [4]. TLR2, in tandem with TLR1 or TLR6, is activated by bacterial cell wall lipopeptides and peptidoglycan. TLR4, in association with proteins CD14 and MD-2, recognizes the lipid A component of lipopolysaccharide (LPS) of most Gram-negative organisms. The importance of TLR2 and TLR4 in pulmonary host defense has been well established [5-9].

As a Gram-negative bacterium, *Bp *has putative ligands for both TLR2 and TLR4 [4]. Wiersinga et al. found increased TLR2 and TLR4 expression and mRNA levels in monocytes and granulocytes of humans with melioidosis as well as activation of these TLRs by *Bp *in vitro [10]. Wiersinga et al. also reported a protective phenotype in TLR2-deficient mice infected intranasally with *Bp*. However, they unexpectedly observed that *Bp *LPS activates TLR2, not TLR4, in transfected HEK293 cells.

In this study, we evaluated the roles of TLR2 and TLR4 in melioidosis using TLR transfection constructs as well as bone marrow-derived macrophages from TLR-deficient mice. We report here that heat-killed *Bp *activates TLR2 in conjunction with TLR1 or TLR6, as well as TLR4. We also show that cytokine production induced by stimulation of primary cells with heat-killed *Bp *is dependent on TLR4 but that cytokine release is augmented in the absence of TLR2. We further demonstrate that *Bp *LPS and *Bp *lipid A are ligands for TLR4, not TLR2.

## Methods

### Preparation of bacteria

*Bp *BP-1, a clinical isolate cultured from a liver abscess of a Vietnamese patient presenting for medical care in Washington state, was grown for 19 hours at 37°C in LB broth. Bacteria were washed twice in sterile PBS and resuspended in PBS. The bacterial slurry was then heat-killed for 60 minutes at 65°C. Bacterial concentration and confirmation of successful killing was determined by quantitative culture on LB agar of the washed and heat-killed slurries, respectively.

### LPS and lipid A isolation and purification

Large-scale BP-1 LPS preparations were isolated using a hot phenol/water extraction method after growth of BP-1 in LB supplemented with 1 mM MgCl_2 _at 37°C [11]. Subsequently, LPS was treated with RNase A, DNase I and proteinase K to ensure purity from contaminating nucleic acids and proteins [12]. Reference strain *Bp *K96243 LPS was kindly provided by Donald Woods at the University of Calgary. BP-1 and K96243 LPS samples were additionally extracted to remove contaminating phospholipids [13] and TLR2 contaminating proteins [14,15]. LPS preparations were determined to have less than 1% contaminating protein as determined by BCA reaction (Pierce, Rockford, IL). Lipid A was isolated after hydrolysis in 1% SDS at pH 4.5 as described [16]. Briefly, 500 μl of 1% SDS in 10 mM Na-acetate, pH 4.5 was added to a lyophilized sample. Samples were incubated at 100°C for 1 hour, frozen, and lyophilized. The dried pellets were resuspended in 100 μL of water and 1 mL of acidified ethanol (100 μL 4 N HCl in 20 mL 95% EtOH). Samples were centrifuged at 5,000 rpm for five minutes. The lipid A pellet was further washed three times in 1 mL of 95% EtOH. The entire series of washes was repeated twice. Samples were resuspended in 500 μL of water, frozen on dry ice and lyophilized. Before use, samples were resuspended in sterile water. Finally, negative ion matrix-assisted laser desorption ionization time-of-flight (MALDI-TOF) mass spectrometry (MS) experiments were performed for the analysis of lipid A preparations to profile structures present in these preparations. Lyophilized lipid A was dissolved with 10 μL 5-chloro-2-mercaptobenzothiazole (CMBT) (Sigma-Aldrich, St. Louis, MO) MALDI matrix in chloroform/methanol, 1:1 (v/v), and then applied (1 μL) onto the sample plate. All MALDI-TOF experiments were performed using a Bruker Autoflex II MALDI-TOF mass spectrometer (Bruker Daltonics Inc., Billerica, MA). Each spectrum was an average of 200 shots. ES Tuning Mix (Agilent, Palo Alto, CA) was used to calibrate the MALDI-TOF MS.

### HEK293 transfections and stimulations

HEK293 cells were cultured in a 96 well flat-bottomed tissue culture plate at ~5 × 10^4 ^cells/well in DMEM plus 10% FBS. The following day cells were transiently transfected with 5 μL of transfection reagent comprised of a 1:1 mix of 0.25 M CaCl_2 _containing 2 × BBS (50 mM BES, 280 mM NaCl, and 1.5 mM NaH_2_PO_4_) and the following DNA: NF-κB-dependent firefly ELAM luciferase and control β-actin-dependent *Renilla *luciferase; and human or murine CD14, MD-2 (except as noted), with either TLR2, TLR2 and TLR1, TLR2 and TLR6, or TLR4 [17]. Two different human TLR1 plasmids were transfected in conjunction with TLR2, one with G at base pair 1805 (which yields lower responses to Pam3CSK4) and one with T at this position (which yields higher responses to Pam3CSK4) [7]. DNA was added in the following amounts to each well: hu- or muMD2 0.0025 μg, hu- or muCD-14 0.0025 μg, ELAM luciferase 0.01 μg, and *Renilla *luciferase 0.0003 μg. When transfected alone, the following amounts of DNA were added: hu- or muTLR2 0.0025 μg, huTLR4 0.0025 μg, or muTLR4 0.0003 μg. When co-transfected with TLR1 or TLR6, huTLR2 0.00125 μg or muTLR2 0.0025 μg was used in addition to huTLR1 0.00125 μg or muTLR1 0.025 μg; or huTLR6 0.0125 μg or muTLR6 0.0025 μg. All transfections were normalized to 0.05 μg total DNA with the addition of empty vector. Transfected cells were washed once after four hours and were stimulated the following day with experimental ligands, non-specific stimulus recombinant human IL-1β (Pierce Endogen, Rockford, IL), control TLR4 ligand ultrapure *Escherichia coli *0111:B4 LPS (Invivogen, San Diego, CA), or control TLR2 ligand Pam3CSK4 (Invivogen and EMC Microcollections, Tuebingen, Germany). After four hours, cells were lysed with passive lysis buffer (Promega, Madison, WI) and NF-κB activation was determined in 10 μL of lysate by the ratio of firefly to *Renilla *luciferase light emission using the Dual Luciferase Reporter System (Promega, Madison, WI).

### Animals

Specific-pathogen-free C57BL/6 mice were obtained from Jackson Laboratories (Bar Harbor, ME). TLR2^-/-^, TLR4^-/-^, TLR2/4^-/-^, and MyD88^-/- ^mice backcrossed at least six generations to C57BL/6 mice were obtained from Dr. Chris Wilson at University of Washington. All animals were housed in laminar flow cages and were permitted ad lib access to sterile food and water. Euthanasia was accomplished with intraperitoneal pentobarbital followed by exsanguination from cardiac puncture prior to bone marrow harvest. The Institutional Animal Care and Use Committee of the University of Washington approved all experimental procedures.

### Bone marrow-derived macrophage stimulations

Femurs and tibias were harvested under sterile conditions from wild type, TLR-deficient, and MyD88^-/- ^mice. Marrow was flushed out using a 26 gauge needle through a 0.2 μm strainer and cultured in Petri dishes in RPMI media supplemented with 1% L-glutamine, 1% penicillin-streptomycin, 10% fetal bovine serum and 20% L929 cell conditioned media at 37°C under 5% CO2 for 5–10 days to allow macrophages to predominate. The monolayer was washed twice with HBSS or media, and macrophages resuspended in RPMI media supplemented with 1% L-glutamine, 1% HEPES, and 10% fetal bovine serum. Cells were added to a 96 well flat-bottomed tissue culture plate at 3 × 10^4 ^to 2 × 10^5 ^cells/well, depending on the experiment. The following day, cells were stimulated with experimental ligands, control TLR4 ligand ultrapure *E. coli *0111:B4 LPS (Invivogen, San Diego, CA), or control TLR2 ligand Pam3CSK4 (Invivogen) in fresh media added to each well. After stimulation for 24 hours, supernatants were removed and stored at -80°C until assayed. TNF-α, MIP-2, and IL-10 were quantified in the supernatants using DuoSet ELISA (R&D Systems, Minneapolis, MN).

### Statistical analyses

Comparisons between two groups of normally distributed data were performed using the t test. Comparisons between three or more groups of normally distributed data were performed with analysis of variance followed by the Bonferroni post-test between groups. Statistical testing was undertaken using Stata v9.0 (StataCorp, College Station, TX). Statistical significance was declared for two-tailed p < 0.05.

## Results

**Figure 1:**
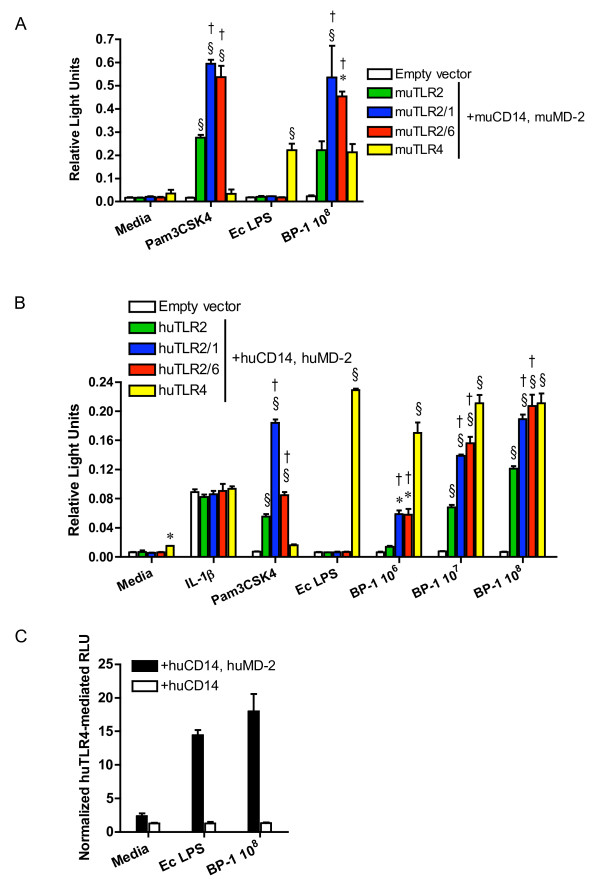
***B. pseudomallei *activates TLR2 and TLR4**. HEK293 cells were transiently transfected with **(A) **murine or **(B) **human TLR2, TLR2/1, TLR2/6, or TLR4; co-receptors CD14 and MD-2; and NF-κB-dependent firefly ELAM luciferase and control β-actin-dependent *Renilla *luciferase. In **(C) **HEK293 cells were transiently transfected with human TLR4 and co-receptor CD14 with or without MD-2; and firefly ELAM and *Renilla *luciferases. Cells were stimulated with media alone, **(B) **IL-1β 20 ng/mL, **(A & B) **Pam3CSK4 100 ng/mL, *E. coli *0111:B4 LPS 10 ng/mL, or heat-killed BP-1 at various concentrations in CFU/mL. NF-κB activation was measured by light emission (relative light units). In **(A&B) **data plotted are means ± standard deviations of triplicate conditions. In **(C) **the means ± standard deviations of triplicate human TLR4-mediated relative light units (normalized to mean empty vector values) are plotted from parallel experiments with or without co-transfection of MD-2. For (A & B) * indicates p < 0.05 and § indicates p = 0.001 compared with empty vector stimulated with the same ligand, and † indicates p < 0.05 compared with TLR2 stimulated with the same ligand using analysis of variance followed by the Bonferroni post-test. Other comparisons are not shown for clarity. The data in **(A) **represent one of two independently performed experiments. The data in **(B) **represent one of three independently performed experiments, but the data displayed, in contrast to the two other experiments, show the response to a hyper-responding variant TLR1 plasmid. The data in **(C) **represent one of three independently performed experiments.

In order to identify TLRs that recognize *Bp*, HEK293 cells transiently transfected with plasmids expressing murine TLR2, TLR2/1, TLR2/6, or TLR4, and co-receptors CD14 and MD-2 were stimulated with a heat-killed *Bp *clinical isolate BP-1 (Figure 1A). Using an NF-κB-dependent luciferase reporter assay, strong *Bp*-induced NF-κB activation was detected in TLR2- and TLR4-transfected cells with augmentation of the TLR2-dependent signal in the presence of TLR1 or TLR6. To determine whether similar signaling is triggered by human receptors in response to *Bp*, HEK293 cells transfected with plasmids expressing human TLRs were also stimulated with *Bp *(Figure 1B). TLR2- and TLR4-mediated NF-κB activation was observed in a largely dose dependent fashion. Both TLR1 and TLR6 augmented the TLR2-mediated response. We also transfected a hypo-responsive variant human TLR1 plasmid in addition to TLR2 in this system and observed little amplification of the TLR2-dependent signal upon stimulation with *Bp *(data not shown). Because MD-2 is a necessary molecule for TLR4 signaling, human TLR4 transfection was repeated without co-transfecting MD-2. In the absence of MD-2, a TLR4-mediated signal in response to *Bp *was not detected (Figure 1C) although TLR2-mediated signaling was not altered (data not shown).

**Figure 2:**
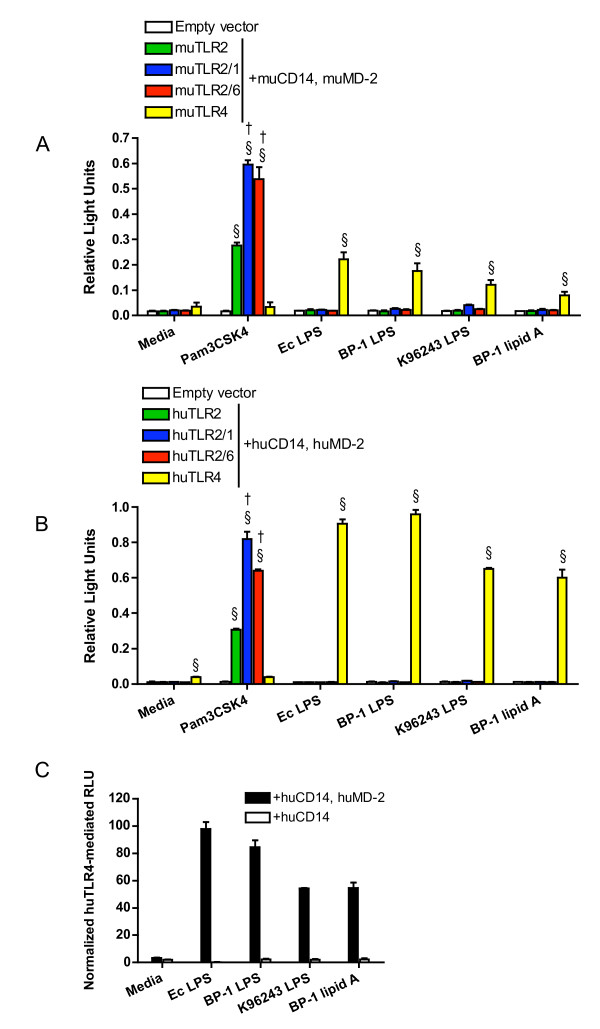
***B. pseudomallei *LPS signals via TLR4**. HEK293 cells were transiently transfected with **(A) **murine or **(B) **human TLR2, TLR2/1, TLR2/6, or TLR4; co-receptors CD14 and MD-2; and NF-κB-dependent firefly ELAM luciferase and control β-actin-dependent *Renilla *luciferase. In **(C) **HEK293 cells were transiently transfected with human TLR4 and co-receptor CD14 with or without MD-2; and firefly ELAM and *Renilla *luciferases. Cells were stimulated with media alone, **(A & B) **Pam3CSK4 1000 ng/mL, *E. coli *0111:B4 LPS 10 ng/mL, BP-1 LPS 10 ng/mL, K96243 LPS 10 ng/mL, or BP-1 lipid A 10 ng/mL. NF-κB activation was measured by light emission (relative light units). In **(A&B) **data plotted are means ± standard deviations of duplicate or triplicate conditions. In **(C) **the means ± standard deviations of triplicate human TLR4-mediated relative light units (normalized to mean empty vector values) are plotted from parallel experiments with or without co-transfection of MD-2. For **(A & B) **§ indicates p < 0.001 compared with empty vector stimulated with the same ligand, and † indicates p < 0.001 compared with TLR2 stimulated with the same ligand using analysis of variance followed by the Bonferroni post-test. Other comparisons are not shown for clarity. Each graph represents one of two independently performed experiments.

Since robust TLR4-dependent NF-κB activation was observed using heat-killed *Bp*, we sought to identify which *Bp *ligand activates TLR4 signaling. As *Bp *LPS was the most likely candidate, LPS was isolated and purified from the *Bp *clinical isolate BP-1. LPS from the *Bp *reference strain K96243 was acquired and also purified. Moreover, because lipid A is the specific component of LPS that activates TLR4, lipid A was isolated from BP-1. HEK293 cells transiently transfected with murine or human TLR2, TLR2/1, TR2/6, or TLR4, and co-receptors CD14 and MD-2 were stimulated with these ligands (Figure 2A &2B). In both murine- and human-transfected cells, robust TLR4-dependent NF-κB activation was identified in response to LPS from both *Bp *strains and in response to BP-1 lipid A without evidence of TLR2 stimulation by these ligands. Additionally, identical stimulations with *Bp *LPS and BP-1 lipid A of cells transfected with human TLR4 and CD14 in the absence of MD-2 were performed, and no TLR4-mediated NF-κB activation was observed (Figure 2C).

**Figure 3:**
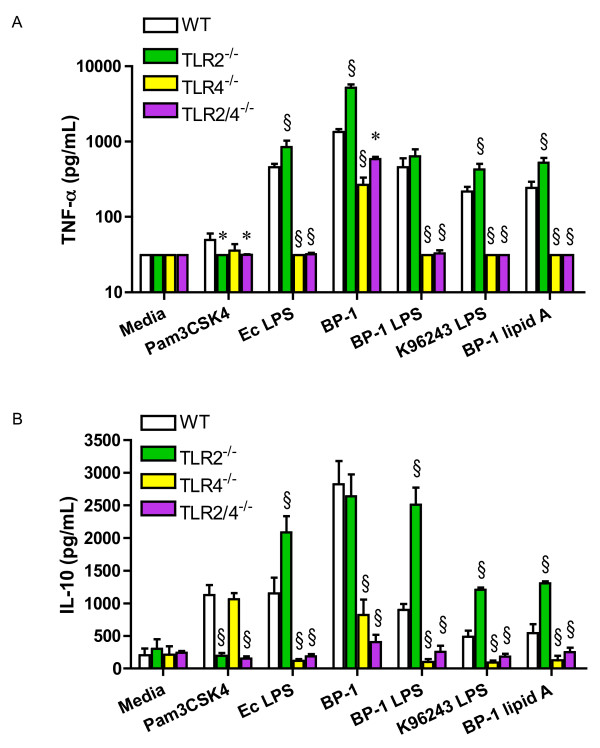
**Absence of TLR4, but not TLR2, impairs the macrophage cytokine response to *B. pseudomallei***. Bone marrow harvested from wild type, TLR2^-/-^, TLR4^-/-^, or TLR2/4^-/- ^mice was cultured in the presence of L929 cell conditioned media for 5–10 days to promote differentiation of macrophages before plating and stimulating with media alone, Pam3CSK4 1000 ng/mL, *E. coli *0111:B4 LPS 100 ng/mL, heat-killed BP-1 at a bacteria to cell ratio of 100, BP-1 LPS 1000 ng/mL, K96243 LPS 1000 ng/mL, or BP-1 lipid A 1000 ng/mL. Supernatants were harvested after 24 hours and **(A) **TNF-α or **(B) **IL-10 production was measured by ELISA. Data plotted are means ± standard deviations of quadruplicate samples. * indicates p < 0.05 and § indicates p = 0.001 compared with wild type cells stimulated with the same ligand, using analysis of variance followed by the Bonferroni post-test. Other comparisons are not shown for clarity. The TNF-α data displayed are from one of six independently performed experiments stimulating various combinations of wild type, TLR2^-/-^, TLR4^-/-^, or TLR2/4^-/- ^macrophages with these ligands, and measuring TNF-α. In one of three experiments comparing cytokine responses from TLR2^-/- ^macrophages to wild type macrophages, production of TNF-α in response to heat-killed *Bp *was not significantly increased. The IL-10 data displayed are from one of five independently performed experiments stimulating various combinations of wild type, TLR2^-/-^, TLR4^-/-^, or TLR2/4^-/- ^macrophages with these ligands, and measuring IL-10. In two of three experiments comparing cytokine responses from TLR2^-/- ^macrophages to wild type macrophages, production of IL-10 in response to heat-killed *Bp *was not significantly increased.

These observations in a transfected cell line prompted us to examine the cytokine response to *Bp *and *Bp *LPS in primary cells. Therefore, bone marrow-derived macrophages from wild type, TLR2^-/-^, TLR4^-/-^, and TLR2/4^-/- ^mice were stimulated with heat-killed BP-1, BP-1 LPS, K96243 LPS, or BP-1 lipid A. Pro-inflammatory cytokine TNF-α was quantified in cell supernatants by ELISA after 24 hours of incubation (Figure 3A). Compared to wild type cells, TNF-α production by TLR4^-/- ^cells in response to BP-1 stimulation was significantly reduced, but was completely eliminated in response to BP-1 LPS, K96243 LPS, or BP-1 lipid A. In TLR2^-/- ^cells, however, TNF-α production after stimulation with BP-1 was either the same as or greater than that produced by wild type cells in several different experiments (3.8 fold greater in the experiment shown). TLR2^-/- ^cells invariably produced more TNF-α than wild type cells after stimulation with BP-1 LPS, K96243 LPS, or BP-1 lipid A (1.4, 2.0, or 2.2 fold greater, respectively, in the experiment shown). Furthermore, TLR2/4^-/- ^cells demonstrated only partial reduction in TNF-α production compared to TLR4^-/- ^cells stimulated with BP-1. Chemokine MIP-2 was also measured in the supernatants of wild type, TLR2^-/-^, TLR4^-/-^, and TLR2/4^-/- ^cells stimulated with the same ligands, and a similar pattern to TNF-α production was observed (data not shown). A higher concentration of *Bp *LPS than control *Escherichia coli *LPS was used in these experiments because in a separate dose response experiment stimulating wild type macrophages, both strains of *Bp *LPS yielded several fold less TNF-α production than similar concentrations of *E. coli *LPS (data not shown).

The findings indicating augmented pro-inflammatory cytokine production in the absence of TLR2 when cells were stimulated with *Bp*, *Bp *LPS, and *Bp *lipid A led us to evaluate whether this was an IL-10-mediated phenomenon. IL-10 is an anti-inflammatory cytokine that may be released by cells upon TLR2 stimulation [18]. We therefore measured IL-10 in cell supernatants stimulated with heat-killed BP-1, BP-1 LPS, K96243 LPS, or BP-1 lipid A (Figure 3B). However, we did not identify any impairment in IL-10 production by TLR2^-/- ^cells in response to these ligands. In general, the overall pattern of IL-10 production was again similar to TNF-α production, with augmentation of BP-1 LPS-, K96243 LPS-, or BP-1 lipid A-induced IL-10 release in the absence of TLR2.

**Figure 4:**
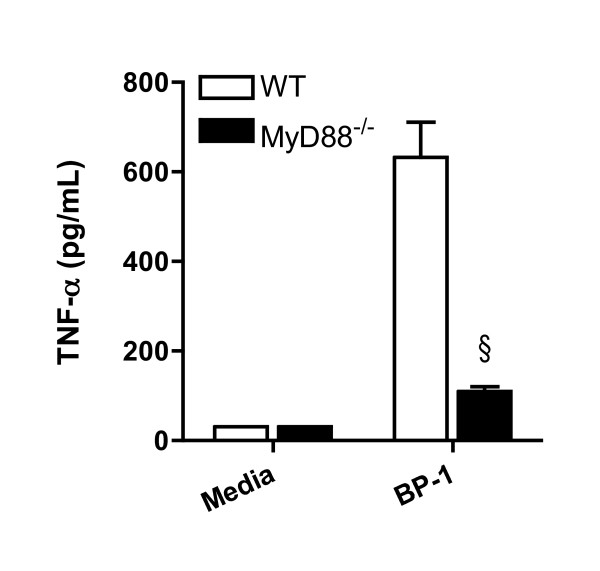
**MyD88 regulates TNF-α production by macrophages stimulated with *B. pseudomallei***. Bone marrow harvested from wild type or MyD88^-/- ^mice was cultured in the presence of L929 cell conditioned media for 5–10 days to promote differentiation of macrophages before plating and stimulating with media alone or heat-killed BP-1 at a bacteria to cell ratio of 100. Supernatants were harvested after 24 hours and TNF-α production was measured by ELISA. Data plotted are means ± standard deviations of quadruplicate samples. § indicates p < 0.001 compared with wild type cells, using the t test. The data displayed represent one of two independently performed experiments.

As the TLR adaptor molecule myeloid differentiation factor 88 (MyD88) mediates both TLR2 and TLR4 signaling, MyD88^-/- ^bone marrow-derived macrophages were therefore stimulated with heat-killed BP-1 and cytokine production assayed after 24 hours (Figure 4). Compared to wild type cells, TNF-α production by MyD88^-/- ^macrophages was markedly curtailed.

## Discussion

In this report, we demonstrate that heat-killed *Bp *activates TLR2 and TLR4. We further show that *Bp *LPS and *Bp *lipid A are TLR4 ligands and we identify augmented macrophage cytokine production induced by *Bp, Bp *LPS or *Bp *lipid A in the absence of TLR2.

TLRs are innate immune receptors that recognize conserved pathogen motifs and initiate an inflammatory signaling cascade. We and others have demonstrated the essential roles of TLRs in host defense against a variety of pulmonary pathogens [5-9]. TLR2 is a promiscuous receptor that generally recognizes bacterial lipotechoic acid, peptidoglycans, or lipoproteins and functions as a heterodimer with either TLR1 or TLR6 [19-22]. LPS is the prototypical bacterial ligand for TLR4 [23] and is comprised of a core polysaccharide, polysaccharide side chains (the O-antigen) and lipid A (endotoxin), a glucosamine-based phospholipid. Lipid A usually interacts with TLR4 in addition to proteins CD14 and MD-2 to induce a pro-inflammatory signal [24]. MD-2 is a soluble molecule that is associated with the extracellular domain of TLR4 and is generally regarded as essential for LPS recognition by TLR4 [25-28]. Both TLR2 and TLR4 are therefore likely to initiate host defense signaling against *Bp*, a Gram-negative pathogen.

As expected, our gain-of-function studies using murine and human constructs confirm a role for TLR2, in conjunction with TLR1 or TLR6, in initiating inflammatory signaling in response to heat-killed *Bp*. As TLR2 forms heterodimers with TLR1 or TLR6, the signal detected after transfection of TLR2 alone is likely explained by low level endogenous expression of TLR1 in HEK293 cells [22,29]. The role of TLR1 in facilitating TLR2-mediated NF-κB activation in response to *Bp *is further elucidated by our observation of minimal augmentation of the TLR2-dependent signal when we transfected a known hypo-responding variant human TLR1 plasmid [17].

We also show that heat-killed *Bp *reliably signals via TLR4 and that *Bp *LPS isolated from two separate bacterial strains activates NF-κB in a uniquely TLR4-mediated fashion. We further demonstrate that *Bp *LPS subcomponent lipid A is a TLR4 ligand in cells transfected with either human or murine TLR constructs. Our observations that *Bp *LPS and lipid A are TLR4 ligands differ from the results of Wiersinga et al. [10], who used HEK293 cells stably transfected with TLR2/CD14 or TLR4/CD14 to show that *Bp *LPS signals through TLR2 but not TLR4. These differences may be explained by our co-transfection of MD-2, a molecule that is not endogenously expressed by HEK293 cells [29], but that we demonstrate is essential for TLR4-dependent signaling in response to *Bp, Bp *LPS, or *Bp *lipid A. While the LPS of a several bacteria signal via TLR2 rather than TLR4 [30-33], some LPS preparations that appear to stimulate TLR2 may be contaminated with lipopeptides [34]. Alternatively, there may be strain-specific differences in *Bp *LPS structure that influence recognition by TLR2 and TLR4. Although our results indicating roles for TLR2 and TLR4 in this gain-of-function system used heat-killed *Bp*, they corroborate recent work with live *Bp *by others [35].

Our studies using primary murine cells provide additional evidence that *Bp *induces a TLR4-mediated inflammatory cytokine response and that *Bp *LPS and lipid A are TLR4 ligands. We demonstrate augmented *Bp*-induced TNF-α production in the absence of TLR2 alone and in the contemporaneous absence of TLR4. This differs from the TLR2-dependent TNF-α production by alveolar macrophages or whole blood stimulated with *Bp *by Wiersinga et al. [10] and may be attributable to the different cell types or bacterial strains used. However, our observations of enhanced cytokine production support their findings of reduced bacterial loads and improved survival in respiratory infection in TLR2^-/- ^mice compared to wild type mice. We also observed consistent augmentation in cytokine production in TLR2^-/- ^macrophages compared to wild type macrophages upon stimulation with *Bp *LPS or with ultrapure *E. coli *LPS, the control ligand. We have observed this phenomenon in previous studies of intracellular TNF-α production by primary bone marrow cells stimulated with *Salmonella minnesota *Re595 LPS, including in the absence of differential TLR7-dependent signaling between wild type and TLR2^-/- ^cells [7]. Others have described a role for TLR2 agonists in suppressing TLR4-mediated inflammatory cytokine production [18] and a role for TLR4 in contributing to pulmonary inflammation in response to the TLR2 ligand lipoteichoic acid [36]. There are also other examples of cooperative TLR2 and TLR4 signaling in host defense [37,38]. However, we are not aware of specific descriptions of the absence of TLR2 influencing the inflammatory response to TLR4-specific stimulation alone. The mechanisms underlying this amplifying effect of TLR2 deficiency upon TLR4-mediated cytokine production by *Bp *LPS are unclear. If the presence of TLR2 is indeed suppressive, our data using LPS suggests that mere expression of the receptor rather than activation may be adequate to modify TLR4-specific signaling. Other reports indicate that TLR2 stimulation may induce production of the anti-inflammatory cytokine IL-10, promoting a Th2-type response [18,39-42]. However, our data do not support this mechanism of TLR2-mediated pro-inflammatory cytokine suppression. To the contrary, we show that *Bp *LPS-induced IL-10 production is augmented in the absence of TLR2, and, despite some differences largely attributable to experimental variability, generally mirrors the pattern of pro-inflammatory cytokine release observed. It may be that in the absence of TLR2, shared signaling molecules are more available to enhance activation of other TLRs, including TLR4. Further investigation will be required to elucidate the interaction of TLR2 and TLR4 in mediating recognition of *Bp*.

Both TLR2 and TLR4 signal via the adaptor molecule MyD88, although TLR4 may also signal in a MyD88-independent fashion via adaptor molecule TIR-domain-containing adapter-inducing interferon-β (TRIF) [43]. Our observations that pro-inflammatory cytokine production by heat-killed *Bp *is largely abolished in the absence of MyD88 whereas TLR2/4^-/- ^cells demonstrate only partial impairment in TNF-α release suggest that other MyD88-dependent receptors may be important in host recognition of *Bp*. Specifically, TLR5 and TLR9, receptors that recognize bacterial flagellin and CpG DNA, respectively, may account for some of this residual MyD88-dependent response. TRIF-mediated TLR4 signaling may also play a role in the innate immune response to *Bp *infection. These additional host defense pathways require further study. Importantly, our experiments have used only heat-killed *Bp *and we cannot discount differential signaling induced by live *Bp*. Thus, additional investigations with live bacteria are warranted.

Our experiments also indicate that *Bp *LPS is a weaker inducer of TNF-α production in bone marrow-derived macrophages than *E. coli *LPS. Others have shown that *Bp *LPS is poorly pyrogenic but more mitogenic compared to *Salmonella abortus equi *[44]. *Bp *LPS may contribute to bacterial pathogenesis by modulating the host response and inhibiting macrophage killing [45]. Although *Bp *LPS is antigenically indistinguishable from the less virulent organism *Burkholderia thailandensis *[46], we have observed that *Bp *LPS may be a stronger inducer of pro-inflammatory cytokine production in macrophages than *B. thailandensis *LPS (unpublished data). Structural analysis of LPS preparations from these *Burkholderia *strains is required.

## Conclusion

*Bp *activates TLR2 and TLR4. In the presence of MD-2, *Bp *LPS and lipid A are TLR4 ligands. Although the macrophage cytokine response to *Bp *LPS or lipid A is completely dependent on TLR4, in TLR2^-/- ^macrophages stimulated with *Bp, Bp *LPS or lipid A, cytokine production is augmented. Other MyD88-dependent signaling pathways may also be important in the host response to *Bp *infection. These findings provide new insights into critical mechanisms of host defense in melioidosis.

## Abbreviations

*Bp: Burkholderia pseudomallei*; LPS: Lipopolysaccharide; MyD88: Myeloid differentiation factor 88; TLR: Toll-like receptor; TRIF: TIR-domain-containing adapter-inducing interferon-β

## Competing interests

The authors declare that they have no competing interests.

## Authors' contributions

TEW, RKE and SJS designed the experiments. RKE extracted LPS and lipid A. MJJ and TEW performed the experiments. TEW and SJS analyzed the data. TEW wrote the manuscript with input from MJJ, RKE and SJS.
